# Differential analysis of virulence and antibiotic resistance genes in ST11 CRKP and ST15 CRKP isolates from a tertiary hospital

**DOI:** 10.3389/fmicb.2026.1787931

**Published:** 2026-03-18

**Authors:** Hui Gao, Yanye Tu, Xingwei Chen, Jiliang Yan, Qiaoping Wu

**Affiliations:** Clinical Laboratory of Ningbo Medical Centre Lihuili Hospital, Ningbo, China

**Keywords:** carbapenem-resistant *Klebsiella pneumoniae*, PLVPK-like virulence plasmid, resistance genes, ST11 CRKP, ST15 CRKP, virulence genes

## Abstract

**Objective:**

To compare the virulence and resistance gene profiles between ST11 and ST15 carbapenem-resistant *Klebsiella pneumoniae* (CRKP) isolates from a hospital in Ningbo, China, and to provide insights for clinical treatment and infection control.

**Methods:**

A total of 129 clinical CRKP isolates were collected and subjected to multilocus sequence typing (MLST) to identify ST11 and ST15 strains. Resistance and virulence genes were detected by PCR, and antimicrobial susceptibility was determined using the VITEK-2 Compact system.

**Results:**

Among the isolates, 48 were ST11 CRKP and 43 were ST15 CRKP. Both groups exhibited high resistance to ertapenem, ciprofloxacin, cefoperazone/sulbactam, imipenem, and levofloxacin. ST15 CRKP showed lower resistance to cotrimoxazole and amikacin. The carriage rate of *bla*_KPC-2_ was higher in ST11 CRKP. Virulence analysis revealed that ST11 CRKP predominantly carried the K2 capsular type, while ST15 CRKP mainly expressed K5. ST11 CRKP also showed higher prevalence of iron transporter (*kfu*), adhesion (*kpn*), and pLVPK-like plasmid genes (*rmpA2*, *peg344*, *iucA*).

**Conclusion:**

ST11 CRKP demonstrates stronger resistance and higher virulence potential compared to ST15 CRKP. However, the detection of a hypervirulent ST15 strain indicates its potential risk for evolution. Strengthening the stewardship of carbapenem use is crucial for controlling CRKP dissemination.

## 1 Introduction

*Klebsiella pneumoniae* (KP) is a Gram-negative, facultatively anaerobic bacterium that frequently causes opportunistic infections such as pneumonia, meningitis, and liver abscesses (Navon-Venezia et al., 2017; Angeles-Solano et al., 2025). Prolonged antibiotic use has led to the emergence of multidrug-resistant strains. In particular, the extensive use of cephalosporins and carbapenems has driven the increasing prevalence of carbapenem-resistant *K. pneumoniae* (CRKP) in clinical settings (Shen et al., 2022; Ding et al., 2023). The dominant sequence types (STs) of CRKP include ST11, prevalent in Asia (especially China), and ST258, prevalent in Europe and North America (Ma et al., 2025). However, the ST profile in China is diversifying, and different *Klebsiella pneumoniae* STs can produce carbapenemases and exhibit high virulence, complicating clinical treatment (Chen et al., 2021). While classical *K. pneumoniae* sequence types (STs) are primarily associated with healthcare-associated infections, some STs, particularly ST11, have been reported to combine carbapenemase production with increased virulence, posing a significant public health threat (Su et al., 2020; Zhang et al., 2020; Shi et al., 2024). The enhancement of both virulence and resistance in *Klebsiella pneumoniae* is often linked to the horizontal transfer of hybrid plasmids carrying corresponding genes (Zhao et al., 2022). The spread of such plasmids can create multidrug-resistant, hypervirulent *K. pneumoniae* (CR-hvKP), posing severe therapeutic challenges. ST15 *K. pneumoniae* is an emerging nosocomial clone reported in China, Spain, and Iran (Sharahi et al., 2021; Feng et al., 2023; Pérez-Palacios et al., 2023). This study collected 129 clinical CRKP isolates, performed MLST to screen for ST11 and ST15 strains, and conducted a comparative analysis of their specimen sources, antimicrobial resistance profiles, and virulence characteristics. The aim is to provide a theoretical basis for resistant pathogen detection and nosocomial infection prevention and control.

## 2 Materials and methods

### 2.1 Source of strains and patient data

A total of 129 non-duplicate clinical CRKP isolates were collected from various sources, including blood, sputum, urine, lavage fluid, and other specimens. The inclusion criterion was imipenem resistance (MIC ≥4 μg/mL) as defined by the Clinical and Laboratory Standards Institute (CLSI) M100-S29 guidelines. The clinical data of patients were collected included age, gender, ward and strain source. This study was approved by the Ethics Committee of Ningbo Medical Center LiHuiLi Hospital, Ningbo University (KY2025SL342-01). As this was a retrospective study using bacterial isolates obtained during routine clinical care, the requirement for informed consent was waived.

### 2.2 Bacterial identification and antimicrobial susceptibility testing

Bacterial identification was performed using both the VITEK 2 Compact system (bioMérieux, France) and EXS3600 MALDI-TOF MS system (Zhongyuan Huiji, China). Antimicrobial susceptibility testing (AST) was conducted using the VITEK 2 Compact system with AST-GN cards, which utilize a miniaturized broth microdilution method. The following antimicrobial agents were tested: Amoxicillin/sulbactam, Aztreonam, Ertapenem, Nitrofurantoin, Trimethoprim-sulfamethoxazole, Ciprofloxacin, Piperacillin/Trizabalat, Gentamicin, Cefazolin, Cepharosporin/sulbactam, Ceftriaxone, Ceftazidime, Cefotetan, Cefepime, Tobramycin, Imipenem, Levofloxacin, and Amikacin. Results were interpreted according to CLSI M100 criteria (2023). *Escherichia coli* ATCC 25922 and *Klebsiella pneumoniae* ATCC 700603 were included as quality control strains in all susceptibility testing assays, and these quality control strains were provided by the Ningbo Clinical Testing Center.

### 2.3 Bacterial DNA extraction

Bacterial DNA was extracted using the boiling extraction method. Briefly, 2–3 fresh colonies of each CRKP isolate were suspended in 200 μL of sterile distilled water in a 1.5 mL microcentrifuge tube. The suspension was thoroughly mixed by vortexing and then heated in a heating block at 100 °C for 10 min. After heating, the samples were immediately cooled on ice for 5 min, followed by centrifugation at 12,000 × *g* for 10 min to pellet cell debris. The supernatant containing genomic DNA was carefully transferred to a new sterile microcentrifuge tube and stored at −20 °C until use. The extracted DNA was used as template for subsequent PCR amplification of antimicrobial resistance and virulence genes.

### 2.4 Detection of antimicrobial resistance and virulence genes

PCR amplification was used to detect carbapenemase resistance genes. The targeted genes included: Class A carbapenemase (*Klebsiella pneumoniae* carbapenemase, KPC); Class B carbapenemases (imipenemase, IMP; New Delhi metallo-β-lactamase, NDM; Verona integron-encoded metallo-β-lactamase, VIM); Class D oxacillinases (OXA-48, OXA-23); efflux pump genes (*AcrAB-TolC*, *oqxB*, *oqxA*); and the disinfectant resistance gene *qacEΔ1-sul1*. Six common capsular polysaccharide genes (K1, K2, K5, K20, K54, K57) and common virulence genes in CRKP. The targeted virulence genes included the capsular polysaccharide synthesis regulator gene (rmpA), siderophore genes (*aerobactin*, *ybtS*, *kfu*, *entB*, *irp-1*, *irp-2*, *fyuA*), pilus-mediated adhesion and colonization genes (*fimH*, *kpn*, *mrkD*), and pLVPK-like virulence plasmid-associated genes (*rmpA2*, *peg-344*, *iroB*, *iucA*) (Jiang et al., 2024). Shanghai Shenggong Biological Engineering Co., LTD synthesized the primer sequences (Supplementary material). All PCR amplifications were performed in a final volume of 25 μL. The reaction mixture contained 1 × PCR buffer (with 1.5 mM MgCl₂), 0.2 mM of each deoxynucleotide triphosphate (dNTP), 0.4 μM of each forward and reverse primer, 1.0 U of Taq DNA polymerase (TaKaRa, Japan), and 50 ng of template DNA. The PCR conditions were as follows: initial denaturation at 94 °C for 5 min; followed by 30 cycles of denaturation at 94 °C for 45 s, annealing at 55 °C for 45 s, and extension at 72 °C for 1 min; with a final extension at 72 °C for 10 min (Jiang et al., 2024).

### 2.5 Multilocus sequence typing (MLST)

PCR amplification was performed for seven housekeeping genes (*gapA*, *infB*, *mdh*, *pgi*, *phoE*, *ropB*, *tonB*). Sequencing of all isolates was conducted according to the protocol provided on the MLST website.^1^ The obtained sequences were compared against the database to determine the sequence types (STs).

### 2.6 PCR amplification product electrophoresis

The PCR products were separated by electrophoresis on a 1.0% (w/v) agarose gel using 1 × Tris-acetate-EDTA (TAE) electrophoresis buffer at 110 V for 15 min. Gels were stained with ethidium bromide, and images were captured using a Gel Doc XR + gel imaging system (Bio-Rad, USA). When confirmation of PCR products by sequencing was required, the target bands were excised from the gel and purified using the QIAquick Gel Extraction Kit (Qiagen, Germany), following the manufacturer’s instructions. The purified products were then sent to Sangon Biotech (Shanghai, China) for sequencing.

### 2.7 Statistical analysis

Data were analyzed using SPSS version 20.0. For comparisons of categorical variables, such as the prevalence of resistance or virulence genes between ST11 and ST15 groups, the Chi-square (*χ*^2^) test or Fisher’s exact test (when expected cell counts were <5) was used. For continuous variables, such as the number of resistance genes per isolate, the Shapiro–Wilk test was first used to test for normality. As the data were not normally distributed, the Mann–Whitney *U* test was employed for comparisons between the two groups. A *p*-value of <0.05 was considered statistically significant.

## 3 Results

### 3.1 Strain sources and basic clinical data

Among 129 CRKP isolates, 48 (37.2%) were ST11 and 43 (33.3%) were ST15. Patient gender distribution was similar between groups (ST11 CRKP: 79.2% male; ST15 CRKP: 69.8% male). The mean age was slightly higher in the ST15 group (69.5 ± 16.1 years) than in the ST11 group (64.1 ± 16.0 years), but the difference was not significant (*p* = 0.066). All ST15 CRKP patients were inpatients, compared to 93.8% of ST11 CRKP patients. Isolates from both groups were primarily from the Neurology department and ICU (ST15 CRKP: 41.9% Neurology, 46.5% ICU; ST11 CRKP: 39.6% Neurology, 37.5% ICU). Sputum was the most common specimen source for both (ST15 CRKP: 55.8%; ST11 CRKP: 60.4%). No significant differences were observed in ward or specimen source distribution (Table 1).

**Table 1:** Strain sources and basic clinical data.

Categories	ST15 CRKP (*N* = 43)	ST11 CRKP (*N* = 48)	*χ*^2^ value	*p* value
Gender				
Male	13 (30.2%)	10 (20.8%)	*χ*^2^ = 0.622	0.43
Female	30 (69.8%)	38 (79.2%)		
Age				
Mean (SD)	69.5 (16.1)	64.1 (16.0)	*W* = 1,264	0.0655
Median [Min, Max]	70.0 [30.0, 94.0]	63 [33.0, 97.0]		
Type of patients				
Outpatient	0 (0%)	3 (6.3%)	*χ*^2^ = 1.16	0.281^a^
Inpatient	43 (100%)	45 (93.8%)		
Ward				
Neurology department	18 (41.9%)	19 (39.6%)	*χ*^2^ = 6.54	0.162^a^
Central ICU	20 (46.5%)	18 (37.5%)		
Pneumology department	2 (4.7%)	6 (12.5%)		
Department of hepatobiliary surgery	2 (4.7%)	0 (0%)		
Others	1 (2.3%)	5 (10.4%)		
Strain source				
Blood	2 (4.7%)	5 (10.4%)	*χ*^2^ = 8.58	0.0726^a^
Sputum	24 (55.8%)	29 (60.4%)		
Urine	8 (18.6%)	7 (14.6%)		
Lavage fluid	0 (0%)	4 (8.3%)		
Others	9 (20.9%)	3 (6.3%)		

a Represents: the Fisher’s exact test (2-sided) is used.

### 3.2 Antimicrobial susceptibility test results of ST15 and ST11 CRKP strains

The antimicrobial susceptibility test results indicated that carbapenem-resistant *Klebsiella pneumoniae* (CRKP) strains exhibited high resistance to various commonly used clinical antimicrobial agents. Both ST15 CRKP and ST11 CRKP strains were completely resistant (100% resistance rate) to drugs including ertapenem, ciprofloxacin, cefoperazone/sulbactam, imipenem, and levofloxacin. However, ST11 CRKP demonstrated a broader resistance profile, with significantly higher resistance rates to certain antimicrobials compared to ST15 strains. Statistical analysis revealed that the resistance rates of ST11 CRKP strains to nitrofurantoin (100% vs. 88.4%, *p* = 0.015), the sulfonamide combination trimethoprim-sulfamethoxazole (64.6% vs. 7.0%, *p* < 0.001), and the aminoglycoside amikacin (93.8% vs. 30.2%, *p* = 0.007) were all significantly higher than those of ST15 CRKP strains (Table 2).

**Table 2:** Susceptibility test results of ST15 CRKP and ST11 CRKP strains.

Antimicrobials	ST15 CRKP (*n* = 43)	ST11 CRKP (*n* = 48)	*χ* ^2^	*p* value
Amoxicillin/sulbactam	40 (93.0%)	44 (91.7%)	0.059	0.808
Aztreonam	37 (86.0%)	44 (91.7%)	0.732	0.392
Ertapenem	43 (100%)	48 (100%)	NA	NA
Nitrofurantoin	38 (88.4%)	48 (100%)	5.906	0.015
Trimethoprim-sulfamethoxazole	3 (7.0%)	31 (64.6%)	32.162	<0.001
Ciprofloxacin	43 (100%)	48 (100%)	NA	NA
Piperacillin/trizabalat	39 (90.7%)	44 (91.7%)	0.027	0.871
Gentamicin	34 (79.1%)	39 (81.3%)	0.068	0.794
Cefazolin	39 (90.7%)	44 (91.7%)	0.027	0.871
Cepharosporin/sulbactam	43 (100%)	48 (100%)	NA	NA
Ceftriaxone	39 (90.7%)	44 (91.7%)	0.027	0.871
Ceftazidime	39 (90.7%)	44 (91.7%)	0.027	0.871
Cefotetan	32 (74.4%)	42 (87.5%)	2.555	0.11
Cefepime	43 (100%)	44 (91.7%)	3.748	0.053
Tobramycin	33 (76.7%)	38 (79.2%)	0.078	0.781
Imipenem	43 (100%)	48 (100%)	NA	NA
Levofloxacin	43 (100%)	48 (100%)	NA	NA
Amikacin	13 (30.2%)	45 (93.8%)	7.235	0.007

NA: not applicable (statistical test could not be performed due to 100% resistance in both groups).

### 3.3 Detection of antimicrobial resistance genes in ST15 and ST11 CRKP

The detection rates of carbapenemase resistance genes *bla*_KPC-2_, *bla*_NDM-1_, and *bla*_OXA-48_ in ST15 CRKP strains were 76.7, 7.0, and 2.3%, respectively. In ST11 CRKP strains, the detection rates for *bla*_KPC-2_ and *bla*_NDM-1_ were 93.8 and 2.1%, respectively. Resistance genes *bla*_IMP-1_, *bla*_VIM-1_, *bla*_VIM-2_, and *bla*_OXA-23_ were not detected in either group. A statistically significant difference was observed between the two groups in the carriage of the *bla*_KPC-2_ resistance gene (*p* < 0.05). Furthermore, the detection rate of the efflux pump gene *oqxA* in ST15 CRKP was 100%, which was higher than that in ST11 CRKP. Conversely, the detection rate of the disinfectant resistance gene *qacEΔ1-sul1* in ST15 CRKP (11.6%) was lower than that in ST11 CRKP, with a statistically significant difference between the two groups (*p* < 0.001) (Table 3).

**Table 3:** Distribution of antimicrobial resistance genes in ST11 and ST15 CRKP isolates.

Antimicrobial resistance genes	ST15 CRKP (*n* = 43)	ST11 CRKP (*n* = 48)	*χ* ^2^	*p* value
*bla*_KPC-2_	33 (76.7%)	45 (93.8%)	4.06	0.044
*bla*_IMP-1_	0 (0%)	0 (0%)	NA	NA
*bla*_NDM-1_	3 (7.0%)	1 (2.1%)	0.39	0.532^a^
*bla*_VIM-1_	0 (0%)	0 (0%)	NA	NA
*bla*_VIM-2_	0 (0%)	0 (0%)	NA	NA
*bla*_OXA-23_	0 (0%)	0 (0%)	NA	NA
*bla*_OXA-48_	1 (2.3%)	0 (0%)	0.00306	0.959^a^
*AcrAB-TolC*	36 (83.7%)	46 (95.8%)	2.5	0.114
*qacE△l-sull*	5 (11.6%)	37 (77.1%)	36.5	<0.001
*oqxB*	9 (20.9%)	5 (10.4%)	1.2	0.273
*oqxA*	43 (100%)	12 (25.0%)	50.3	<0.001

a Represents: the Fisher’s exact test (2-sided) is used; NA: not applicable (statistical test could not be performed due to 100% resistance in both groups).

### 3.4 Detection of capsular serotypes and virulence genes in ST15 and ST11 CRKP

Regarding the distribution of capsular serotypes, ST15 CRKP was predominantly of the K5 type (83.7%), whereas ST11 CRKP was primarily of the K2 type (43.8%), showing a highly significant difference (*p* < 0.001). In terms of key virulence genes, the carriage rates of the siderophore gene *kfu* (89.6% vs. 7.0%, *p* < 0.001) and the pilus adhesion gene *fimK* (91.7% vs. 2.3%, *p* < 0.001) were significantly higher in ST11 CRKP compared to ST15 CRKP, indicating a generally higher prevalence of virulence genes in ST11 strains. Notably, genes associated with the pLVPK-like virulence plasmid were overwhelmingly dominant in ST11 CRKP. The rates of carriage for *rmpA2* (77.1% vs. 27.9%, *p* < 0.001), *peg-344* (70.8% vs. 14.0%, *p* < 0.001), and *iucA* (87.5% vs. 20.9%, *p* < 0.001) were all significantly higher in ST11 strains compared to ST15 strains (Table 4).

**Table 4:** Distribution of virulence genes in ST11 and ST15 CRKP isolates.

Virulence genes	ST15 CRKP (*n* = 43)	ST11 CRKP (*n* = 48)	*χ* ^2^	*p* value
The capsule gene				
K1	0 (0%)	0 (0%)	NA	NA
K2	4 (9.3%)	21 (43.8%)	13.508^a^	<0.001^a^
K5	34 (79.1%)	3 (6.3%)	49.849^a^	<0.001^a^
K20	5 (11.6%)	4 (8.3%)	0.030	0.862^a^
K54	0 (0%)	2 (4.2%)	NA	0.496^a^
K57	0 (0%)	1 (2.1%)	NA	1
Capsule-forming gene				
*rmpA*	0 (0%)	0 (0%)	NA	NA
Iron carrier genes				
*aero*	0 (0%)	0 (0%)	NA	NA
*ybtS*	41 (95.3%)	45 (93.8%)	<0.001	1
*entB*	42 (97.7%)	42 (87.5%)	2.03	0.154
*kfu*	3 (7.0%)	43 (89.6%)	58.7	<0.001^a^
*irp-1*	43 (100%)	48 (100%)	NA	NA
*irp-2*	43 (100%)	46 (95.8%)	0.406	0.524
*fyuA*	42 (95.3%)	47 (97.9%)	<0.001	1
Mucin colonization and adhesion genes				
*fimH*	37 (86.0%)	38 (79.2%)	0.342	0.559
*kpn*	1 (2.3%)	44 (91.7%)	68.9	<0.001^a^
*mrkD*	43 (100%)	48 (100%)	NA	NA
pLVPK-like virulence plasmid				
*rmpA2*	12 (27.9%)	37 (77.1%)	20.1	<0.001
*peg-344*	6 (14.0%)	34 (70.8%)	27.5	<0.001
*iroB*	1 (2.3%)	4 (8.3%)	0.632	0.427^a^
*iucA*	9 (20.9%)	42 (87.5%)	38.1	<0.001

a Represents: the Fisher’s exact test (2-sided) is used; NA: not applicable (statistical test could not be performed due to 100% resistance in both groups).

### 3.5 Correlation between resistance genes and virulence genes in ST15 CRKP and ST11 CRKP

**Figure 1:**
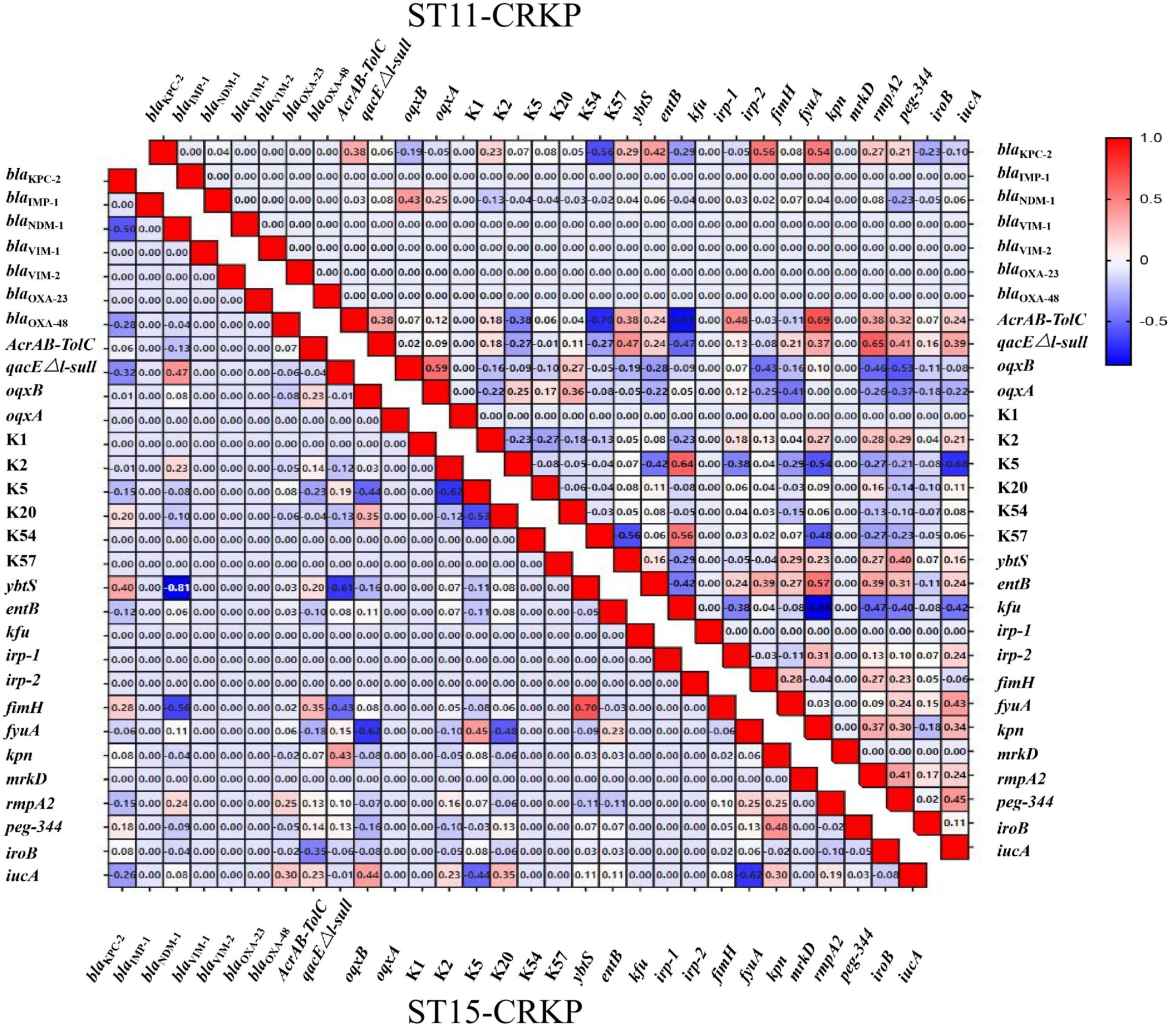
Heatmap of correlations between resistance genes and virulence genes in ST15-CRKP (lower panel) and ST11-CRKP (upper panel). Values indicate correlation coefficients, with 1 and −1 representing perfect positive and negative correlations, respectively.

We analyzed the distribution and correlation between resistance genes and virulence genes (Figure 1). In ST15 CRKP, a highly positive correlation was observed between the *ybtS* gene and *fyuA*, while *ybtS* showed negative correlations with *bla*_NDM-1_ and *qacEΔ1-sul1*. The *iucA* gene exhibited a negative correlation with the *fimH* gene. In ST11 CRKP, the *AcrAB-TolC* gene demonstrated a highly positive correlation with *kpn* but negative correlations with K57 and *kfu*. The *qacEΔ1-sul1* gene showed a positive correlation with *rmpA2*. Furthermore, the K5 gene exhibited a highly positive correlation with *kfu* and a negative correlation with *iucA*.

## 4 Discussion

Our data identify ST11 and ST15 as the predominant CRKP clones in our center, consistent with recent epidemiological shifts in China. While ST11 has been historically dominant, the near-equal proportion of ST15 (47.3%) highlights its emergence as a significant nosocomial threat, as reported globally (Berglund et al., 2019; Feng et al., 2023). Isolates were primarily from sputum of ICU and neurology patients, reflecting common colonization sites and the vulnerability of critically ill, immunocompromised hosts undergoing invasive procedures and broad-spectrum antibiotic therapy.

The antimicrobial susceptibility profiles revealed near-universal resistance to core antimicrobial classes in both groups. However, the significantly higher resistance of ST11 CRKP to amikacin and trimethoprim-sulfamethoxazole has direct clinical implications. This suggests that amikacin may retain utility in ST15 CRKP infections but is unlikely to be effective against ST11 clones, possibly due to co-carriage of 16S rRNA methyltransferase genes (Yang et al., 2011).

Antimicrobial resistance gene analysis identified *bla*_KPC-2_ as the predominant carbapenemase, with a higher prevalence among ST11 strains than ST15 strains. A clear divergence in auxiliary resistance determinants was observed between the two sequence types. The *oqxA* efflux pump gene was universally present in ST15 CRKP isolates, whereas the disinfectant resistance gene *qacEΔ1-sul1* was highly prevalent in ST11 CRKP isolates. These findings highlight distinct resistance gene profiles associated with each clonal lineage. The latter may confer a survival advantage in hospital environments, facilitating persistent colonization and clonal dominance in ICUs (Han et al., 2025). The ubiquitous *oqxA* in ST15 strains warrants monitoring for potential efflux-mediated resistance to agents like tigecycline. In this study, high-level resistance to nitrofurantoin was observed in both ST11 and ST15 CRKP isolates. The near-universal resistance to nitrofurantoin in these strains is not unexpected. As carbapenem-resistant bacteria, they have been selected under the pressure of broad-spectrum antibiotics, while nitrofurantoin is rarely used in settings such as ICUs (Bader et al., 2017); therefore, the selective pressure to maintain susceptibility is weak. Concurrently, the chromosomal mutations that mediate resistance carry a low fitness cost, which may facilitate the persistence of these strains even after the drug is discontinued (Vogwill and MacLean, 2015). From a clinical perspective, this high resistance rate implies that nitrofurantoin is largely ineffective against urinary tract infections caused by these CRKP clones, further emphasizing the importance of performing urine culture and susceptibility testing prior to treatment (Osei Sekyere and Amoako, 2017).

Traditionally, bacterial evolution was thought to be constrained by a fitness trade-off between resistance and virulence, where the metabolic cost of maintaining resistance genes could attenuate pathogenic potential—a concept known as the “resistance-virulence balance” (Fuzi, 2016). Our findings, however, reveal that ST11 CRKP and ST15 CRKP are pursuing distinct evolutionary strategies that appear to challenge or differently engage with this paradigm.

ST11 CRKP seems to be adopting a “high-risk, high-gain” strategy by converging multidrug resistance with hypervirulence. The high carriage rates of pLVPK-like plasmid genes (*rmpA2*, *iucA*, *peg-344*) and the hypervirulent-associated K2 capsule indicate active acquisition and stabilization of large virulence plasmids alongside a robust resistance repertoire (Jia et al., 2024; Nguyen et al., 2025). This suggests that in ST11, the presumed fitness cost of virulence may be offset by the selective advantages conferred in certain niches—for instance, the ability to cause disseminated infections could enhance transmission. Moreover, the positive correlation we observed between the disinfectant resistance gene (*qacEΔ1-sul1*) and virulence markers like *rmpA2* implies possible genetic linkage or co-selection on shared mobile genetic elements. This co-carriage facilitates the emergence of the feared “superbug” phenotype: a pathogen that is difficult to treat, persistent in the hospital environment, and capable of causing severe disease.

In contrast, ST15 CRKP appears to follow a more “specialized” path, prioritizing broad-spectrum antimicrobial resistance—exemplified by the universal presence of the *oqxA* efflux pump—while largely maintaining a lower-virulence profile (predominantly K5, low carriage of key virulence plasmid genes). This aligns with the classical CRKP model and may reflect an ecological strategy optimized for persistence in high-antibiotic-pressure hospital settings, where resistance is the primary determinant of fitness. The observed significant negative correlation between the siderophore gene *ybtS* and disinfectant resistance genes in ST15 isolates is intriguing but does not demonstrate a causal relationship. This association could be due to a variety of factors, such as underlying genetic differences in the ST15 background, co-selection dynamics with other genes, or unmeasured environmental variables. Further functional studies and comparative genomic analyses with a larger sample set are needed to explore the biological basis of this correlation.

The convergence of resistance and virulence in ST11 CRKP represents a grave clinical threat, as it can lead to invasive infections with limited therapeutic options and high mortality, even in immunocompetent hosts (Paczosa and Mecsas, 2016). Although the hypervirulent ST15 strain identified in this study represents a sporadic occurrence rather than a prevailing trend, it serves as a proof-of-concept that the genetic convergence observed in ST11 is theoretically transferable to other lineages, particularly via the hybrid plasmids known to circulate in *K. pneumoniae* populations (Zhao et al., 2022). Continuous genomic surveillance is warranted to monitor if such rare events become more common. The presence of *bla*_KPC-2_ in both clones provides a common resistance platform upon which virulence plasmids could be accreted, especially under intense antimicrobial selection.

Antibiotic selective pressure, particularly the heavy use of carbapenems and other broad-spectrum agents in ICUs, is a primary driver for the enrichment and maintenance of multidrug-resistant clones like ST11 and ST15 (Cienfuegos-Gallet et al., 2022). Hospital environments serve as crucial reservoirs. Studies have shown that CRKP, including ST11 and ST15, can persist on environmental surfaces and equipment, creating opportunities for transmission and ongoing exposure to selective pressures like biocides. This environmental persistence could contribute to the co-selection of resistance and tolerance mechanisms. The convergence of resistance and virulence, particularly in ST11, may be influenced by the plasmid landscape. Recent global plasmidome analyses suggest that specific plasmid types can co-localize in the same bacterial host, facilitating the transfer and stabilization of both resistance and virulence traits under selective pressure (Heng et al., 2025). Weaker or sporadic selective pressures in the hospital environment for ST15 might explain why the convergence of these traits is less stable and thus more rarely observed.

## 5 Conclusion

In conclusion, this study reveals distinct genetic profiles in ST11 and ST15 CRKP from our hospital, with ST11 exhibiting a significantly higher burden of both antimicrobial resistance and virulence genes. These findings underscore the critical need for clone-specific surveillance to monitor the dissemination of high-risk clones like ST11. Furthermore, our results highlight the importance of rigorous antimicrobial stewardship to mitigate the selective pressures driving the maintenance of MDR strains, and strengthened infection control practices to prevent the cross-transmission of these successful lineages within the hospital environment. The sentinel finding of virulence genes in an ST15 isolate also warrants continued vigilance for the convergence of these traits in diverse genetic backgrounds.

## Data Availability

The original contributions presented in the study are included in the article/Supplementary material, further inquiries can be directed to the corresponding authors.

## Footnotes

1
https://bigsdb.pasteur.fr/klebsiella/primers-used
