# Correction: Differential analysis of virulence and antibiotic resistance genes in ST11 CRKP and ST15 CRKP isolates from a tertiary hospital

**DOI:** 10.3389/fmicb.2026.1870635

**Published:** 2026-05-15

**Authors:** Hui Gao, Yanye Tu, Xingwei Chen, Jiliang Yan, Qiaoping Wu

**Affiliations:** Clinical Laboratory of Ningbo Medical Centre Lihuili Hospital, Ningbo, China

**Keywords:** carbapenem-resistant *Klebsiella pneumoniae*, PLVPK-like virulence plasmid, resistance genes, ST11 CRKP, ST15 CRKP, virulence genes

Affiliation Clinical Laboratory of Ningbo Medical Centre Lihuili Hospital, Ningbo, China was erroneously given as Clinical Laboratory of Ningbo Medical Centre Lihuili Hospital, Ningbo University, Ningbo, China.

The original version of this article has been updated.

