# Supplementary material for: Differential analysis of virulence and antibiotic resistance genes in ST11 CRKP and ST15 CRKP isolates from a tertiary hospital
Source: Front Microbiol. 2026 Mar 18;17:1787931. doi: 10.3389/fmicb.2026.1787931 (PMC13039009; doi:10.3389/fmicb.2026.1787931)
Supplement: Supplementary file 1 [file Data_Sheet_1.pdf]

Supplemental material

Supplementary Table 1 Sequences of the pcr primers

Gene	Primer sequences (5'-3')	Product （bp）	Annealing （℃）
rmpA	F: ACTGGGCTACCTCTGCTTCA<br>R: CTTGCATGAGCCATCTTTCA	516	55
rmpA2	F: CTTTATGTGCAATAAGGATGTT<br>R: CCTCCTGGAGAGTAAGCATT	451	52
iucA	F：AATCAATGGCTATTCCCGCTG<br>R：CGCTTCACTTCTTTCACTGACAGG	239	59
iroB	F：ATCTCATCATCTACCCTCCGCTC<br>R：GGTTCGCCGTCGTTTTCAA	235	58
peg-344	F：CTTGAAACTATCCCTCCAGTC<br>R：CCAGCGAAAGAATAACCCC	508	56
irp-1	F: TGAATCGCGGGTGTCTTATGC<br>R: TCCCTCAATAAAGCCCACGCT	238	55
irp-2	F: AAGGATTCGCTGTTACCGGAC<br>R: TCGTCGGGCAGCGTTTCTTCT	287	53
aerobactin	F: GCATAGGCGGATACGAACAT<br>R: CACAGGGCAATTGCTTACCT	556	53
ybtS	F: GACGGAAACAGCACGGTAAA<br>R: GAGCATAATAAGGCGAAAGA	242	50
fyuA	F:GCGACGGGAAGCGATGATTTA<br>R: TAAATGCCAGGTCAGGTCACT	547	57
mrkD	F: CCACCAACTATTCCCTCGAA<br>R: ATGGAACCCACATCGACATT	240	54
fimH	F: ATGAACGCCTGGTCCTTTGC<br>R: GCTGAACGCCTATCCCCTGC	688	56
kfu	F: GAAGTGACGCTGTTTCTGGC<br>R: TTTCGTGTGGCCAGTGACTC	797	58
entB	F: GTCAACTGGGCCTTTGAGCCGGTC<br>R: TATGGGCGTAAACGCCGGTGAT	400	59
kpn	F: GTATGACTCGGGGAAGATTA<br>R: CAGAAGCAGCCACCACACG	626	55
K1	F: GGTGCTCTTTACATCATTGC<br>R: GCCCAGGTTAATGAATCCGT	1283	55

K2	F: GACCCGATATTCATACTTGACAGAG<br>R: CCTGAAGTAAAATCGTAAATAGAT	641	53
K5	F: GCCACCTCTAAGCATATAGC<br>R: CGCACCAGTAATTCCAACAG	540	55
K20	F: CGGTGCTACAGTGCATCATT<br>R: GTTATACGATGCTCAGTCGC	741	56
K54	F: CATTAGCTCAGTGGTTGGCT<br>R: GCTTGACAAACACCATAGCAG	881	55
K57	F: CGACAAATCTCTCCTGACGA<br>R: CGCGACAAACATAACACTCG	1037	50
KPC-2	F: GCTACACCTAGCTCCACCTTC<br>R: ACAGTGGTTGGTAATCCATGC	989	59
NDM-1	F: CTTCCAACGGTTTGATCGTC<br>R: ATTGGCATAAGTCGCAATCC	271	56
IMP-1	F: CTACCGCAGCAGAGTCTTTG<br>R: AACCAGTTTTGCCTTACCAT	587	56
VIM-1	F：AGTGGTGAGTATCCGACAG<br>R: ATGAAAGTGCGTGGAGAC	261	55
VIM-2	F: ATGTTCAAACTTTTGAGTAAG<br>R: CTACTCAACGACTGAGCG	801	55
OXA-23	F: GATCGGATTGGAGAACCAGA<br>R: ATTTCTGACCGCATTTCCAT	501	59
OXA-48	F: TTGGTGGCATCGATTATCGG<br>R: GAGCACTTCTTTTGTGATGGC	744	55
AcrAB-TolC	F: ATGCAAAATTATTCGCTTTCAGGC<br>R: GATACACACCGCCTCCTCAAA	420	72
qacE△l-sull	F: TAGCGAGGGCTTTACTACTAAGC<br>R: ATTCAGAATGCCGAACACCG	300	62
oqxA	F: CTCGGCGCGATGATGCT<br>R: CCACTCTTCACGGGAGACGA	392	55
oqxB	F: TTCTCCCCCGGCGGGAAGTAC<br>R:CTCGGCCATTTTGGCGCGTA	512	56
